# Supplementary material for: Biodiversity of β-Carboline Profile of Banisteriopsis caapi and Ayahuasca, a Plant and a Brew with Neuropharmacological Potential
Source: Plants (Basel). 2020 Jul 9;9(7):870. doi: 10.3390/plants9070870 (PMC7411993; doi:10.3390/plants9070870)
Supplement: Supplementary file 1 [file plants-09-00870-s001.pdf]

Biodiversity of β-carboline profile of *Banisteriopsis caapi* and ayahuasca, a plant and a brew with neuropharmacological potential

Supplementary Material

Spectrometric data for the synthetized N,N dimethyltryptamine (DMT) and tetrahydroharmine (THH):

DMT ^{1}H NMR spectrum: δ 2.34 (6H, s, N(C**H**$_3$)$_2$; 2.61 (2H, m, C**H**$_2$CH$_2$N(CH$_3$)$_2$); 2.92 (2H, m, CH$_2$C**H**$_2$N(CH$_3$)$_2$); 7,0 (1H, m, Ph); 7.08 to 7.20 (2H, m, Ph); 7.32 (1H, m, Ph); 7.59 (1H, m, Ph); 8,11 (1H, s, N-**H**). DMT ^{13}C NMR spectrum: δ 23,70; 45,48; 60,33; 111,11; 114,36; 118,79; 119,17; 121,44; 121,92; 127,47; 136,26. Exact mass [M+H]$^+$: 189.1389.

THH ^{1}H NMR spectrum: δ 1.42 (3H, d, NHCHC**H**$_3$); 2.65 (1H, d m, **H**CHCH$_2$NH); 2.73 (1H, m, HC**H**CH$_2$NH); 3.01 (2H, m, *CH$_2$CH$_2$NH*); 3.33 (1H, m, NHC**H**CH$_3$); 3.83 (3H, s, OC**H**$_3$); 6.76 (1H, d d, Ph); 6.83 (1H, d, Ph); 7.33 (1H, d, Ph). THH ^{13}C NMR spectrum: 20,81; 22,81; 42,84; 48,21; 55,82; 95,08; 108,40; 108,79; 118,62; 122,09; 135,95; 136,38; 156,21. The exact mass determined was [M+H]$^+$ 217.1332.

Table S1. Samples of *Banisteriopsis* ssp. and *Diplopterys pubipetala* and β-carboline concentrations.

Sample Identification	Species/Common Name	Collecting State	Cultivated or Native	Collecting Date	Harmine (mg/g)	Harmaline (mg/g)	THH (mg/g)
JS237*	*B. caapi*/tucunacá	DF	Cultivated	06/24/2016	1.517	1.536	29.037
JS235*	*D. pubipetala*	GO	Native	06/07/2016	<LOD	<LOD	<LOD
JS238*	*B. caapi*/tucunacá	DF	Cultivated	06/24/2016	6.347	0.312	1.580
JS239*	*B. caapi*/tucunacá	DF	Cultivated	06/24/2016	0.083	0.252	3.120
JS242*	*B. caapi*/caupuri	DF	Cultivated	06/24/2016	4.704	0.094	0.843
JS243*	*B. caapi*/tucunacá	DF	Cultivated	06/2016	<LOD	0.463	12.354
JS244*	*B. caapi*/caupuri	DF	Cultivated	08/2016	11.827	1.000	5.264
JS245*	*B. caapi*/caupuri	DF	Cultivated	08/2016	6.807	0.239	1.898
BS134/17	*B. caapi*/tucunacá	DF	Cultivated	01/02/2017	1.525	0.143	8.136
BS137/17	*B. caapi*/caupuri	DF	Cultivated	09/02/2017	2.741	0.353	12.402
RCO3326*	*B. caapi*/tucunacá	DF	Cultivated	06/10/2017	7.663	1.330	3.973
BS163/17	*B. caapi*/tucunacá	AC	Native	11/17/2017	18.269	1.081	4.051
BS164/17	*B. caapi*/tucunacá	AC	Native	11/17/2017	2.264	0.629	18.471
BS165/17	*B. caapi*/tucunacá	AC	Native	11/17/2017	1.398	0.505	7.760
BS166/17	*B. caapi*/tucunacá	AC	Native	11/17/2017	1.225	0.912	13.919
BS167/17	*B. caapi*/tucunacá	AC	Native	11/18/2017	8.332	1.011	3.259
CF2479*	*D. pubipetala*	DF	Native	08/22/2017	<LOD	<LOD	<LOD
CF2467*	*D. pubipetala*	DF	Native	08/22/2017	<LOD	<LOD	<LOD
CF2469*	*D. pubipetala*	DF	Native	08/22/2017	<LOD	<LOD	<LOD
CF2471*	*B. laevifolia*	DF	Native	08/22/2017	<LOD	<LOD	<LOD
CF2470*	*B. laevifolia*	DF	Native	08/22/2017	<LOD	<LOD	<LOD
RCO3392*	*B. muricata*	AC	Native	12/06/2017	<LOD	<LOD	<LOD
JS353*	*Banisteriopsis* sp.	GO	Native	08/11/2017	<LOD	<LOD	<LOD
CF2474*	*B. variabilis*	DF	Cultivated	08/23/2017	<LOD	<LOD	<LOD
CF2435*	*B. megaphylla*	DF	Native	07/04/2017	<LOD	<LOD	<LOD
CF2431*	*B. gardneriana*	DF	Native	07/05/2017	<LOD	<LOD	<LOD
JS341*	*B. oxyclada*	GO	Native	06/30/2017	<LOD	<LOD	<LOD
CF2482*	*B. laevifolia*	DF	Native	01/29/2018	<LOD	<LOD	<LOD

Sample Identification	Species/Common Name	Collecting State	Cultivated or Native	Collecting Date	Harmine (mg/g)	Harmaline (mg/g)	THH (mg/g)
CF2483*	*B. laevifolia*	DF	Native	01/29/2018	<LOD	<LOD	<LOD
CF2454*	*D. pubipetala*	DF	Native	03/26/2018	0.158	0.046	1.037
JS380*	*B. caapi*/ourinho	AC	Cultivated	08/02/2018	<LOD	<LOD	<LOD
JS381*	*Banisteriopsis* sp.	AC	Native	08/02/2018	<LOD	<LOD	<LOD
JS382*	*B. caapi*/ourinho	AC	Native	08/04/2018	<LOD	<LOD	<LOD
JS383*	*B. caapi*/ourinho	AC	Native	08/04/2018	13.580	0.805	2.306
JS384*	*B. caapi*/tucunacá with nodes	AC	Native	08/06/2018	10.303	0.273	1.196
JS385*	*B. caapi*/tucunacá	AC	Native	08/06/2018	10.084	0.490	1.789
JS386*	*B. caapi*/tucunacá with nodes	AC	Native	08/06/2018	12.077	0.844	3.026
JS387*	*B. caapi*/tucunacá	AC	Native	08/06/2018	10.917	0.609	1.549
JS388*	*B. caapi*/tucunacá	AC	Native	08/06/2018	9.948	0.445	1.705
JS389*	*B. caapi*/tucunacá	AC	Native	08/06/2018	5.475	0.400	1.166
JS390*	*B. caapi*/tucunacá	AC	Native	08/06/2018	6.290	1.573	2.895
JS391*	*B. caapi*/amarelinho	AC	Native	08/06/2018	8.539	1.594	3.375
JS392*	*B. caapi*/caboquinho	AC	Native	08/07/2018	0.124	0.001	0.109
RCO3490*	*B. caapi*/tucunacá	GO	Cultivated	07/23/2018	0.754	0.686	1.269
RCO3494*	*B. caapi*/ourinho	AC	Native	08/02/2018	3.829	0.683	1.930
DR130/18	*B. caapi*/ourinho	GO	Cultivated	09/07/2018	6.917	1.583	3.084
AC31/18	*B. caapi*/tucunacá	AC	Native	08/03/2018	3.334	0.158	0.236
AC32/18	*B. caapi*/tucunacá	AC	Native	08/03/2018	4.822	0.494	1.200
AC33/18	*B. caapi*/tucunacá	AC	Native	08/04/2018	6.400	0.434	2.892
AC34/18	*B. caapi*/tucunacá	AC	Native	08/04/2018	4.438	1.609	2.891
AC35/18	*B. caapi*/tucunacá	AC	Native	08/04/2018	2.892	0.424	0.946
AC36/18	*B. caapi*/tucunacá	AC	Native	08/04/2018	0.377	0.005	0.012
AC37/18	*B. caapi*/tucunacá	AC	Native	08/04/2018	0.128	0.009	0.012
AC38/18	*B. caapi*/tucunacá	AC	Native	08/04/2018	8.070	0.355	1.154
AC39/18	*B. caapi*/tucunacá	AC	Native	08/04/2018	0.184	<LOD	<LOD
AC40/18	*B. caapi*/tucunacá	AC	Native	08/04/2018	4.972	0.747	1.734
AC41/18	*B. caapi*/tucunacá	AC	Native	08/04/2018	<LOD	<LOD	<LOD
AC42/18	*B. caapi*/tucunacá	AC	Native	08/04/2018	4.689	1.453	3.738

Sample Identification	Species/Common Name	Collecting State	Cultivated or Native	Collecting Date	Harmine (mg/g)	Harmaline (mg/g)	THH (mg/g)
AC43/18	*B. caapi*/tucunacá	AC	Native	08/04/2018	8.827	0.432	1.930
AC44/18	*B. caapi*/tucunacá	AC	Native	08/04/2018	0.325	0.013	0.065
AC45/18	*B. caapi*/tucunacá	AC	Native	08/04/2018	7.355	0.165	0.959
AC46/18	*B. caapi*/tucunacá	AC	Native	08/04/2018	4.830	0.176	0.416
AC47/18	*B. caapi*/tucunacá	AC	Native	08/04/2018	6.583	0.858	2.998
AC48/18	*B. caapi*/tucunacá	AC	Native	08/04/2018	9.122	0.987	3.373
AC49/18	*B. caapi*/tucunacá	AC	Native	08/04/2018	8.114	0.132	1.196
AC50/18	*B. caapi*/tucunacá	AC	Native	08/04/2018	7.292	0.637	2.182
AC51/18	*B. caapi*/tucunacá	AC	Native	08/04/2018	8.794	0.818	2.855
AC52/18	*B. caapi*/tucunacá	AC	Native	08/06/2018	10.754	0.457	0.793
AC53/18	*B. caapi*/tucunacá	AC	Native	08/06/2018	8.730	0.475	0.831
AC54/18	*B. caapi*/tucunacá	AC	Native	08/06/2018	7.479	0.357	0.513
AC55/18	*B. caapi*/tucunacá	AC	Native	08/06/2018	5.521	0.126	0.069
AC56/18	*B. caapi*/tucunacá	AC	Native	08/06/2018	10.222	0.412	1.271
AC57/18	*B. caapi*/tucunacá	AC	Native	08/06/2018	3.574	0.209	0.122
AC58/18	*B. caapi*/tucunacá	AC	Native	08/06/2018	5.623	0.326	0.506
AC59/18	*B. caapi*/tucunacá	AC	Native	08/06/2018	0.969	0.171	0.713
AC60/18	*B. caapi*/tucunacá	AC	Native	08/06/2018	4.443	0.188	1.106
AC61/18	*B. caapi*/tucunacá	AM	Native	08/07/2018	6.955	0.650	2.290
AC62/18	*B. caapi*/tucunacá	AM	Native	08/07/2018	11.079	0.231	1.067
AC63/18	*B. caapi*/tucunacá	AM	Native	08/07/2018	3.283	0.365	3.850
AC64/18	*B. caapi*/tucunacá	AM	Native	08/07/2018	2.324	0.663	7.874
AC65/18	*B. caapi*/tucunacá	RO	Native	08/08/2018	2.380	0.187	0.760
AC66/18	*B. caapi*/tucunacá	RO	Native	08/08/2018	2.320	0.875	13.132
AC67/18	*B. caapi*/tucunacá	RO	Native	08/08/2018	2.320	0.875	13.132
AC68/18	*B. caapi*/tucunacá	RO	Native	08/08/2018	1.989	0.756	6.818
AC69/18	*B. caapi*/tucunacá	AC	Native	08/15/2018	2.872	0.347	3.215
AC70/18	*B. caapi*/tucunacá	AC	Native	08/15/2018	6.785	0.575	1.513
RD01/18	*B. caapi*/caupuri	RO	Native	08/19/2018	<LOD	<LOD	<LOD
RD02/18	*B. caapi*/caupuri	RO	Native	08/19/2018	<LOD	<LOD	<LOD

Sample Identification	Species/Common Name	Collecting State	Cultivated or Native	Collecting Date	Harmine (mg/g)	Harmaline (mg/g)	THH (mg/g)
RD03/18	*B. caapi*/caupuri without nodes	RO	Native	08/19/2018	2.060	0.255	1.033
RD04/18	*B. caapi*	RO	Native	07/06/2018	2.636	0.727	12.526
RD05/18	*B. caapi*	RO	Native	07/06/2018	6.727	0.218	1.215
RD11/18	*B. caapi*/tucunacá	RO	Native	09/05/2018	2.478	0.597	9.696
RD12/18	*B. caapi*/tucunacá	RO	Native	09/05/2018	6.650	0.215	0.387
RD13/18	*B. caapi*/tucunacá	RO	Native	09/05/2018	5.886	0.164	0.256
RD14/18	*B. caapi*/tucunacá	RO	Native	09/07/2018	5.979	0.152	0.175
RD15/18	*B. caapi*/tucunacá	RO	Native	09/07/2018	6.746	0.351	1.430
RD16/18	*B. caapi*/tucunacá	RO	Native	09/02/2018	3.710	0.349	1.001
RD17/18	*B. caapi*/tucunacá	RO	Native	08/02/2018	5.904	0.732	2.067
RD18/18	*B. caapi*/tucunacá	RO	Native	09/07/2018	5.164	0.092	0.270
RD19/18	*B. caapi*/tucunacá	RO	Native	09/07/2018	6.325	0.351	1.132
RD20/18	*B. caapi*/tucunacá	RO	Native	09/07/2018	6.045	0.280	0.842
RD31/18	*B. caapi*/amarelinho	RO	Native	08/20/2018	4.820	0.260	3.481
RD32/18	*B. caapi*/amarelinho	RO	Native	08/20/2018	4.351	0.093	0.146
RD33/18	*B. caapi*/amarelinho	RO	Native	08/12/2018	7.936	0.324	1.125
RD34/18	*B. caapi*/amarelinho	RO	Native	08/24/2018	7.226	0.564	1.646
RD35/18	*B. caapi*/amarelinho	RO	Native	08/20/2018	<LOD	<LOD	<LOD
RD36/18	*B. caapi*/amarelinho	RO	Native	08/14/2018	6.088	0.767	2.351
RD37/18	*B. caapi*/amarelinho	RO	Native	08/18/2018	4.731	0.236	0.268
RD38/18	*B. caapi*/amarelinho	RO	Native	08/24/2018	5.547	0.297	1.424
RD39/18	*B. caapi*/amarelinho	RO	Native	09/03/2018	1.285	<LOD	<LOD
AC01/18	*B. caapi*/tucunacá	AC	Native	08/15/2018	0.284	<LOD	<LOD
AC02/18	*B. caapi*/tucunacá	AC	Native	08/15/2018	0.618	0.004	0.010
AC03/18	*B. caapi*/tucunacá	AC	Native	08/15/2018	7.189	0.143	0.102
AC04/18	*B. caapi*/tucunacá	AC	Native	08/15/2018	1.864	<LOD	<LOD
AC05/18	*B. caapi*/tucunacá	AC	Native	08/15/2018	0.627	0.004	<LOD
AC06/18	*B. caapi*/tucunacá	AC	Native	08/18/2018	10.696	0.900	1.444
AC07/18	*B. caapi*/tucunacá	AC	Native	08/18/2018	<LOD	<LOD	<LOD
AC08/18	*B. caapi*/tucunacá	AC	Native	08/18/2018	10.966	0.409	1.305

Sample Identification	Species/Common Name	Collecting State	Cultivated or Native	Collecting Date	Harmine (mg/g)	Harmaline (mg/g)	THH (mg/g)
AC09/18	*B. caapi*/tucunacá	AC	Native	08/18/2018	6.032	0.109	0.340
AC10/18	*B. caapi*/tucunacá	AC	Native	08/18/2018	11.908	0.949	2.006
AC11/18	*B. caapi*/tucunacá	AC	Native	09/07/2018	0.415	<LOD	<LOD
AC12/18	*B. caapi*/tucunacá	AC	Native	09/07/2018	0.694	<LOD	<LOD
AC13/18	*B. caapi*/tucunacá	AC	Native	09/07/2018	2.963	0.234	0.656
AC14/18	*B. caapi*/tucunacá	AC	Native	09/07/2018	1.207	0.043	0.080
AC15/18	*B. caapi*/tucunacá	AC	Native	09/07/2018	1.994	<LOD	<LOD
AC17/18	*B. caapi*/tucunacá	AC	Native	09/13/2018	9.234	0.416	0.393
AC18/18	*B. caapi*/tucunacá	AC	Native	09/13/2018	7.111	0.488	1.009
AC19/18	*B. caapi*/tucunacá	AC	Native	09/13/2018	3.420	0.239	0.262
AC20/18	*B. caapi*/tucunacá	AC	Native	09/13/2018	13.873	0.606	1.708
AC21/18	*B. caapi*/tucunacá	AC	Native	09/02/2018	4.133	0.146	0.406
AC22/18	*B. caapi*/tucunacá	AC	Native	09/02/2018	3.502	0.267	2.050
AC23/18	*B. caapi*/tucunacá	AC	Native	09/02/2018	0.171	<LOD	<LOD
AC24/18	*B. caapi*/tucunacá	AC	Native	09/02/2018	4.200	0.301	1.300
AC25/18	*B. caapi*/tucunacá	AC	Native	09/02/2018	4.820	0.273	0.345
AC26/18	*B. caapi*/tucunacá	AM	Native	08/15/2018	5.729	0.165	0.849
AC27/18	*B. caapi*/tucunacá	AM	Native	08/15/2018	15.951	1.244	0.842
AC28/18	*B. caapi*/tucunacá	AM	Native	08/16/2018	6.834	0.660	0.804
AC29/18/18	*B. caapi*/tucunacá	AM	Native	08/17/2018	10.886	0.626	1.286
AC30/18	*B. caapi*/tucunacá	AM	Native	08/18/2018	13.720	2.076	4.818
DF01/19	*B. caapi*/tucunacá	GO	Cultivated	01/24/2019	7.746	0.677	1.448
RCO3674*	*B. caapi*/tucunacá	PA	Cultivated	07/09/2019	4.596	0.736	2.361
RCO3675*	*B. caapi*/tucunacá	PA	Cultivated	07/09/2019	7.300	1.513	3.833
RCO3677*	*B. caapi*/tucunacá	PA	Cultivated	07/09/2019	2.932	0.335	0.955
RCO3680*	*B. caapi*/caupuri	PA	Cultivated	07/09/2019	1.830	0.406	0.756
RCO3682*	*B. caapi*/caupuri	PA	Native	07/09/2019	10.374	1.514	4.223
RCO3684*	*B. caapi*/caupuri	PA	Native	07/09/2019	0.750	0.151	0.153
RCO3687*	*B. caapi*/tucunacá	PA	Cultivated	07/10/2019	2.635	0.377	0.392
RCO3689*	*B. caapi*/tucunacá	PA	Cultivated	07/10/2019	3.979	0.663	1.760

Sample Identification	Species/Common Name	Collecting State	Cultivated or Native	Collecting Date	Harmine (mg/g)	Harmaline (mg/g)	THH (mg/g)
RCO3690*	*B. caapi*/tucunacá	PA	Cultivated	07/10/2019	3.486	0.408	1.223
RCO3691*	*B. caapi*/tucunacá	PA	Cultivated	07/10/2019	6.711	0.308	0.666
RCO 3697*	*B. caapi*/caupuri	PA	Cultivated	07/10/2019	<LOD	<LOD	<LOD
RCO3698*	*B. caapi*/caupuri	PA	Cultivated	07/10/2019	6.768	1.305	3.584
RCO3702*	*B. caapi*/tucunacá	PA	Cultivated	07/10/2019	1.217	0.248	0.272
RCO3707*	*B. caapi*/tucunacá	PA	Native	07/11/2019	1.671	0.149	0.355
RCO3709*	*B. caapi*/tucunacá	PA	Cultivated	07/11/2019	4.253	1.181	2.829
RCO3710*	*B. caapi*/tucunacá	PA	Cultivated	07/11/2019	2.039	0.471	0.939
RCO3712*	*B. caapi*/caupuri	PA	Cultivated	07/11/2019	2.134	0.275	0.464
CB37*	*B. caapi*/caupuri	AM	Cultivated	08/23/2019	2.421	0.497	1.051
CB38*	*B. caapi*/caupuri	AM	Cultivated	08/23/2019	1.273	0.138	0.375
CB39*	*B. caapi*/ourinho	AM	Cultivated	08/23/2019	1.015	0.193	0.704
CB40*	*B. caapi*/caupuri	AM	Cultivated	08/23/2019	<LOD	<LOD	<LOD
CB41*	*B. caapi*/pajezinho	AM	Cultivated	08/23/2019	0.407	0.076	0.338
CB42*	*B. caapi*/caupuri	AM	Cultivated	08/25/2019	1.684	0.265	1.046
CB43*	*B. caapi*/caupuri	AM	Cultivated	08/25/2019	2.966	0.614	1.435
CB45*	*B. caapi*/ourinho	AM	Cultivated	08/25/2019	0.899	0.253	0.694
CB47*	*B. caapi*/caupuri	AM	Cultivated	08/25/2019	1.053	0.448	0.528
CB50*	*Banisteriopsis* sp.	AM	Cultivated	08/25/2019	<LOD	<LOD	<LOD
RCO3516*	*B. caapi*/tucunacá	MG	Cultivated	12/27/2019	3.287	0.614	1.948
RCO3519*	*B. caapi*/tucunacá	MG	Cultivated	12/27/2019	1.937	0.228	0.623
RCO3553*	*B. caapi*/quebrador	DF	Cultivated	03/12/2019	5.229	0.619	1.901
RCO3619*	*B. caapi*/ourinho	DF	Cultivated	04/04/2019	2.498	0.590	1.424
RCO3643*	*B. caapi*/tucunacá	DF	Cultivated	04/17/2019	1.734	0.205	0.657
RCO3620*	*B. caapi*/ourinho	DF	Cultivated	04/04/2019	1.025	0.108	0.170
RCO3621*	*B. caapi*/ourinho	DF	Cultivated	04/04/2019	1.634	0.321	0.493
LC156/19	*B. caapi*/caupuri without nodes	GO	Cultivated	12/05/2019	10.370	0.093	0.301
LC157/19	*B. caapi*/amarelinho	GO	Cultivated	12/05/2019	6.774	0.329	0.268

* Samples stored in the UB herbarium and UBw wood collection of the University of Brasilia; RO=Rondonia; AC=Acre; DF= Federal District; GO=Goiás, MG=Minas Gerais; AM=Amazonas; PA=Pará; LOD = 0.0009 mg/g for harmine, 0.0018 mg/g for harmaline and 0.0146 mg/g for tetrahydroharmine.

Table S2. Ayahuasca brew samples and alkaloid concentrations.

Sample	Collection Date	Ayahuasca Group/State	Observation	DMT (mg/mL)	Harmina (mg/mL)	Harmalina (mg/mL)	THH (mg/mL)
BS136/17	01/02/2017	UDV/DF	Prepared with tucunacá in 2017	0.096	0.669	0.084	1.095
BS140/17	09/02/2017	UDV/DF	Prepared with tucunacá in 2017	0.279	0.986	0.224	1.406
BS143/17	06/10/2017	CELF/DF	Prepared with tucunacá in 2017	0.537	1.133	0.288	2.363
BS172/17	11/18/2017	UDV/AC	Prepared with tucunacá in 2017	0.304	0.788	0.035	0.346
RCO01/17	11/18/2017	Daime/AC	Honey prepared in 2015	2.513	2.434	0.310	2.981
RCO02/17	11/18/2017	Daime/AC	Honey prepared in 2014	1.616	2.063	0.209	1.995
RCO03/17	11/18/2017	Daime/AC	Prepared with ourinho in 2015	0.773	0.704	0.085	0.788
RCO04/17	11/18/2017	Daime/AC	Honey prepared with "arara" in 2015	1.470	1.043	0.062	0.810
RCO05/17	11/18/2017	Daime/AC	Prepared with ourinho in 2015	0.600	0.327	0.049	0.490
RCO06/17	11/18/2017	Daime/AC	Prepared with ourinho in 2015	1.324	1.283	0.111	1.009
RCO07/17	11/18/2017	Daime/AC	Prepared with ourinho in 2015	1.755	0.163	0.045	0.559
RCO08/17	11/18/2017	Daime/AC	Honey prepared with 2 parts vine and 1 part *P. viridis* in 2015	0.870	1.463	0.119	1.039
RCO09/17	11/18/2017	Daime/AC	honey prepared with 2 parts vine and 1 part *P. viridis* in 2015	0.131	0.109	0.012	0.086
RCO10/17	11/18/2017	Daime/AC	Prepared with arara in 2015	2.111	2.081	0.212	2.063
RCO11/17	11/18/2017	Daime/AC	Honey prepared with ourinho in 2015	1.459	1.309	0.208	1.339
RCO12/17	11/18/2017	Daime/AC	Prepared with 2 parts ourinho and 1 part *P. viridis in 2015*	0.956	0.142	0.020	0.344
RCO13/17	11/18/2017	Daime/AC	Prepared with arara and ourinho in 2015	1.088	0.632	0.108	1.073
RCO14/18	04/06/2018	Daime/AC	Prepared with tucunacá only in 2018	<LOD	0.140	0.026	0.269
RCO15/18	04/06/2018	Daime/GO	Prepared with tucunacá, ourinho and caupuri in 2018	1.384	0.818	0.173	1.114
RCO16/18	04/06/2018	Daime/GO	"Brew of the names" prepared with tucunacá in 2018.	1.646	0.896	0.178	1.114
RCO17/18	04/30/2018	Daime/GO	honey prepared with tucunacá and diluted with the vine in 2018	1.001	0.870	0.145	0.990
DR224/18	09/19/2018	ICEFLU/GO	Prepared with tucunacá in 2018	0.881	1.088	0.219	1.770
VF02/19	07/01/2018	Daime/AC	Prepared in 2018	1.429	1.043	0.078	0.848

Sample	Collection Date	Ayahuasca Group/State	Observation	DMT (mg/mL)	Harmina (mg/mL)	Harmalina (mg/mL)	THH (mg/mL)
DF04/19	01/25/2019	Daime/GO	Prepared with tucunacá in 2019	1.699	0.722	0.136	1.421
RCO18/19	01/15/2019	Daime/DF	Almost honey prepared with tucunacá in 2018	0.791	0.660	0.093	0.938
RCO19/19	01/15/2019	Daime/DF	Prepared with tucunacá in 2018	0.733	0.546	0.073	0.874
RCO20/19	04/06/2018	Daime/DF	honey prepared with tucunacá in 2017	1.249	1.016	0.195	1.219
LC158/19	08/12/2019	UDV/GO	Prepared with caupuri without nodes (14.3%) and amarelinho (85.7%) in 2019	0.483	1.084	0.073	0.449
CB01/20	02/10/2020	Daime/DF	Prepared in 2019	0.900	2.989	0.701	1.395
CB02/20	02/10/2020	Daime /DF	Prepared in 2017	0.549	2.029	0.468	1.114
CB03/20	02/13/2020	Daime /DF	Prepared in 2019	3.120	7.110	0.945	3.053
CB04/20	02/01/2020	Daime /AC	Prepared in 2019	0.365	1.279	0.324	0.998
LC06/20	02/11/2020	UDV/AC	Prepared with tucunacá in 2020	0.556	2.681	0.440	0.949

honey is a term used when the brew is concentrated through further boiling. DF= Federal District, AC = Acre, GO=Goias, UDV=União do Vegetal; ICEFLU= Igreja do Culto Eclético da Fluente Luz Universal; LOD = 0.0006 mg/mL.

Table S3. Optimized ESI+ MS/MS parameters, chromatographic retention times and ion ratios in the LC-MS/MS method for the target analytes in *B. caapi* extracts and ayahuasca.

**Analyte Structure**	**DP (V)**	**Transition (*m*/*z*)**	**CE (V)**	**CXP (V)**	**RT, min**	**Ion Ratio (RSD, %)***
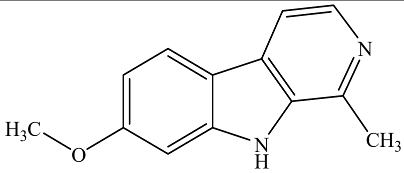 Harmine; $[M+H]^+ = 213$	86	170<br>198	43<br>33	12<br>16	6.4	1.24<br>(1.03)
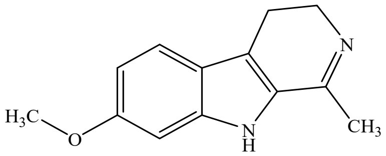 Harmaline $[M+H]^+ = 215$	71	174<br>200	33<br>33	14<br>14	5.8	1.01<br>(2.30)
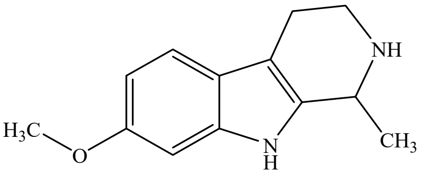 THH $[M+H]^+ = 217$	46	188<br>200	17<br>19	14<br>16	3.4	1.35<br>(1.99)
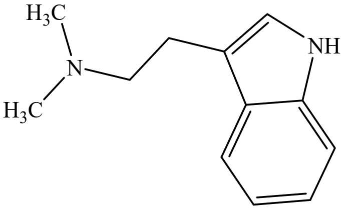 DMT; $[M+H]^+ = 189$	41	144<br>143	25<br>45	10<br>8	2.3	5.58<br>(1.18)

DP = declustering potential; CE = collision energy; CXP = collision cell exit potential; RT = retention time * quantifier/qualifier obtained through the validation experiments (*n* = 70); RSD: relative standard deviation.

Table S4. Recovery and repeatability (*n* = 3 for DMT and *n* = 5 for the others) and intermediate precision (*n* = 8) obtained for the analysis of Malpighiaceae extracts and ayahuasca samples at three concentration levels.

Analyte	Level (mg/g; mg/mL)*	Recovery, Mean (%)	Repeatability (RSD %)	Intermediate Precision (RSD %)
Harmine	0.75; 0.005	100.5	11.4	14.5
	3.00; 0.02	88.6	5.9	7.4
	7.50; 0.05	92.3	7.5	8.5
Harmaline	0.075; 0.0005	91.8	5.8	8.1
	0.375; 0.025	98.2	2.8	7.9
	1.50; 0.1	90.7	9.0	9.2
THH	0.75; 0.005	94.6	11.3	9.5
	3.00; 0.02	89.5	6.4	12.3
	7.50; 0.05	87.9	8.2	11.6
DMT	-; 0.005	103.1	7.1	-
	-; 0.02	98.7	1.5	-
	-; 0.05	92.1	11.4	-

RSD = relative standard deviation. * level at Malpighiaceae extract; level adjusted for ayahuasca sample; underlined values are the limit of quantification of the method.
